# A Rare Case of Mucinous Carcinoma Arising in Association with an Intraductal Papilloma

**DOI:** 10.70352/scrj.cr.25-0446

**Published:** 2026-03-04

**Authors:** Haruka Yamasaki, Sayaka Kuba, Momoko Akashi, Michi Morita, Aki Yukutake, Yuki Hara, Aya Tanaka, Hajime Imamura, Ryota Otsubo, Megumi Matsumoto, Kengo Kanetaka, Keitaro Matsumoto, Susumu Eguchi, Rin Yamaguchi

**Affiliations:** 1 Department of Surgery, Nagasaki University Graduate School of Biomedical Sciences, Nagasaki, Nagasaki, Japan; 2 Department of Breast and Endocrine Surgery, Nagasaki Harbor Medical Center, Nagasaki, Nagasaki, Japan; 3 Department of Breast and Endocrine Surgery, National Hospital Organization Nagasaki Medical Center, Omura, Nagasaki, Japan; 4 Department of Surgical Oncology, Nagasaki University Graduate School of Biomedical Sciences, Nagasaki, Nagasaki, Japan; 5 Department of Breast Center/Diagnostic Pathology, Nagasaki University Hospital, Nagasaki, Nagasaki, Japan

**Keywords:** mucinous carcinoma, intraductal papilloma, breast cancer, transition zone

## Abstract

**INTRODUCTION:**

Intraductal papilloma (IDP) is a benign breast lesion that accounts for 3%–6% of core biopsy diagnoses. It is considered a high-risk precursor due to its association with atypia, ductal carcinoma in situ, and invasive carcinoma. Although IDP-NOS (not otherwise specified) rarely progresses to invasive cancer, IDP with atypia carries a higher risk of malignant transformation.

**CASE PRESENTATION:**

A 57-year-old woman presented with a mass in the left breast. Mammography revealed a microlobulated mass, and ultrasonography showed a well-defined, coarse isoechoic mass measuring 26 mm with a disrupted anterior border. MRI revealed a high T2 signal outside the center of the tumor, with early enhancement and a plateau phase. Based on these imaging findings, mucinous carcinoma was suspected; a core needle biopsy revealed both mucinous carcinoma and ductal carcinoma with a mucocele-like lesion. The patient subsequently underwent a total mastectomy and sentinel lymph node biopsy for cT2N0 breast cancer. The surgical specimen revealed a mucinous carcinoma spreading around the papilloma, with a distinct transition zone from papilloma to mucinous carcinoma.

**CONCLUSIONS:**

This case suggests that mucinous carcinoma may arise from an intraductal papilloma, emphasizing the importance of appropriate clinical follow-up.

## INTRODUCTION

Intraductal papilloma (IDP) is a benign tumor found within breast ducts, it occurs in perimenopausal women within an age range between 30 and 50 years.^1)^ It comprises approximately 3%–6% of breast core biopsy diagnoses.^2)^ IDP is classified as a high-risk precursor lesion due to its association with atypia, ductal carcinoma in situ (DCIS), and invasive carcinoma.^3)^ Histologically, IDP is classified as IDP without atypia (IDP-NOS [not otherwise specified]) and IDP with atypical ductal hyperplasia (ADH).^4)^ Atypical papilloma (IDP with atypia) is defined as a papilloma containing a monomorphic epithelial proliferation with architectural and cytologic features of low-grade DCIS,^1)^ involving less than one-third of the lesion.^5)^ According to the 5th edition of the WHO Classification of Breast Tumours, the distinction between ADH and low-grade DCIS is now based on lesion size, with lesions <2 mm classified as ADH and those ≥2 mm as DCIS.^4)^ A meta-analysis of the upgrade of IDP to cancer reported that 5% of IDP-NOS and 36% of IDP with ADH are upgraded to carcinomas.^6)^ IDP-NOS was reported to rarely progress to invasive cancer, whereas 15.9% of IDP with ADH transition to invasive carcinoma.^6)^ Among the lesions diagnosed as IDP on biopsy but progressed to invasive carcinoma after surgical excision, invasive ductal carcinoma (IDC) was the most common, accounting for 80%, followed by solid papillary carcinoma (12.8%), invasive lobular carcinoma (5.1%), and encapsulated papillary carcinoma (2.6%).^6)^ Here, we report the case of a 57-year-old woman who was suspected of having transitioned from an IDP to a mucinous carcinoma.

## CASE PRESENTATION

**Fig. 1:**
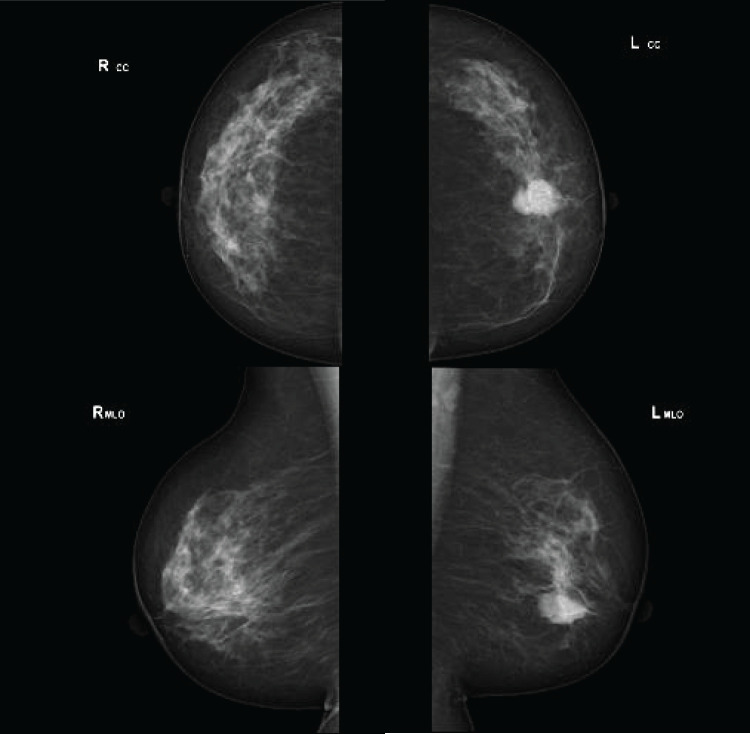
Mammography showing a microlobulated mass in the left breast, classified as BI-RADS category 4. BI-RADS, Breast Imaging Reporting and Data System

**Fig. 2:**
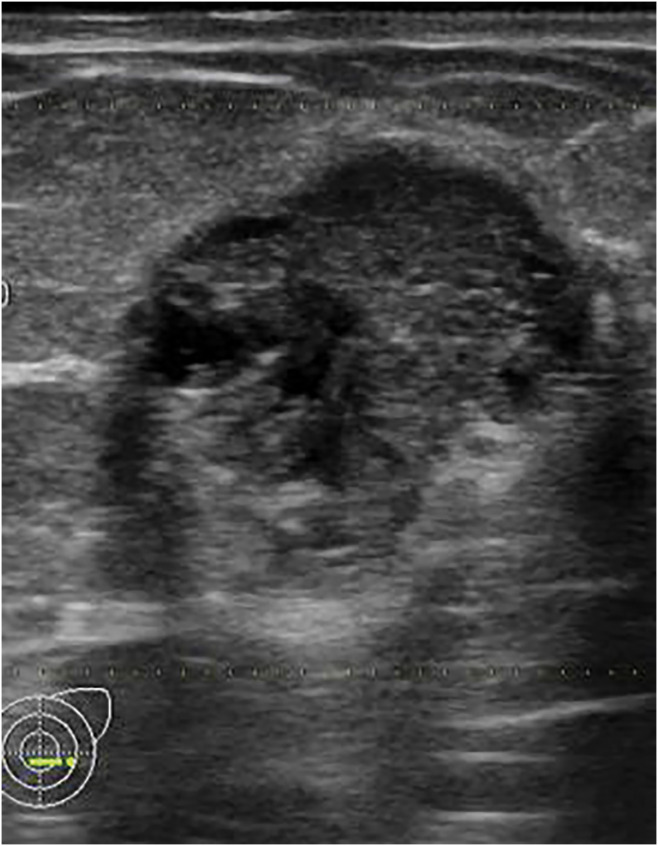
Ultrasonography showing a circumscribed isoechoic tumor measuring 26 mm with enhanced posterior echo.

**Fig. 3:**
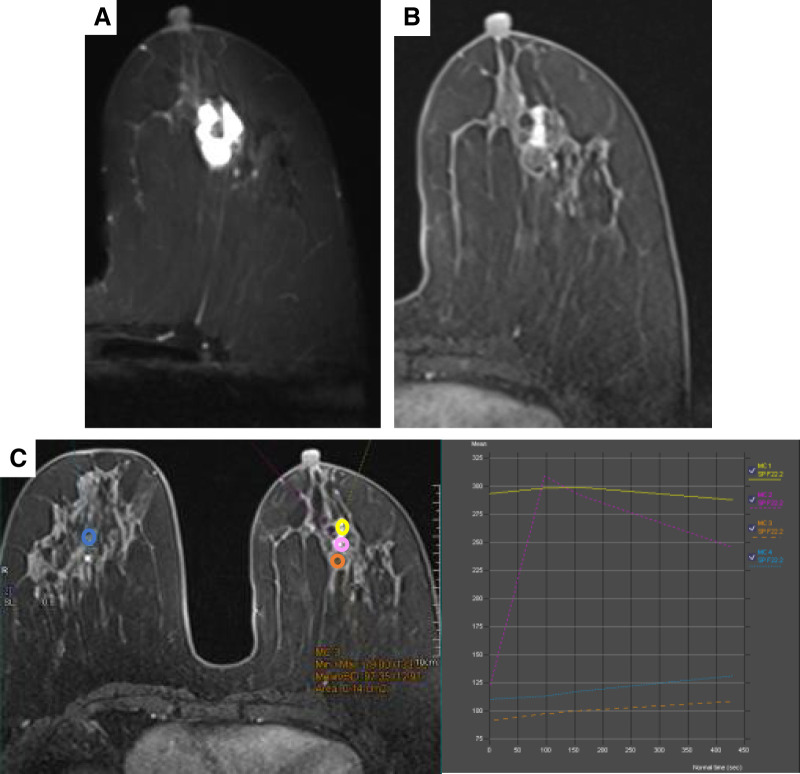
(**A**) In the T2-weighted MRI, areas other than the center exhibit high signal intensity. (**B**) On dynamic MRI, contrast enhancement is observed in the central part of the tumor. (**C**) The time–intensity curve of the enhanced central region (yellow, orange, and pink) exhibits different enhancement patterns, including rapid plateau and normal patterns.

A 57-year-old female patient presented to our hospital with a 2-month history of a left breast mass. She had never undergone breast cancer screening and had no family history of breast or ovarian cancers. On physical examination, a 3-cm mass was palpated in the left breast, without swelling of the axillary lymph nodes. Mammography revealed a microlobulated, high-density mass in the left breast, classified as Breast Imaging Reporting and Data System (BI-RADS) category 4 (**Fig. 1**). Ultrasonography revealed a circumscribed isoechoic tumor measuring 26 mm, with an enhanced posterior echo (**Fig. 2**). The tumor was associated with a halo and a ruptured anterior border, increasing the suspicion of invasive carcinoma. MRI revealed a high T2 signal except at the center of the tumor (**Fig. 3A**). Contrast enhancement was observed in the central part of the tumor on dynamic contrast-enhanced MRI (**Fig. 3B**). The time–intensity curve at the center demonstrated a rapid plateau pattern (**Fig. 3C**). Based on these findings, a mucinous carcinoma mixed with invasive breast carcinoma of no special type in the central part was suspected. A core needle biopsy revealed mucinous and ductal carcinomas with mucocele-like lesions. The Nottingham histological grade was 1, and the tumor was positive for estrogen receptor (ER), and progesterone receptor (PgR), but negative for human epidermal growth factor receptor type 2 (HER2) expression.

**Fig. 4:**
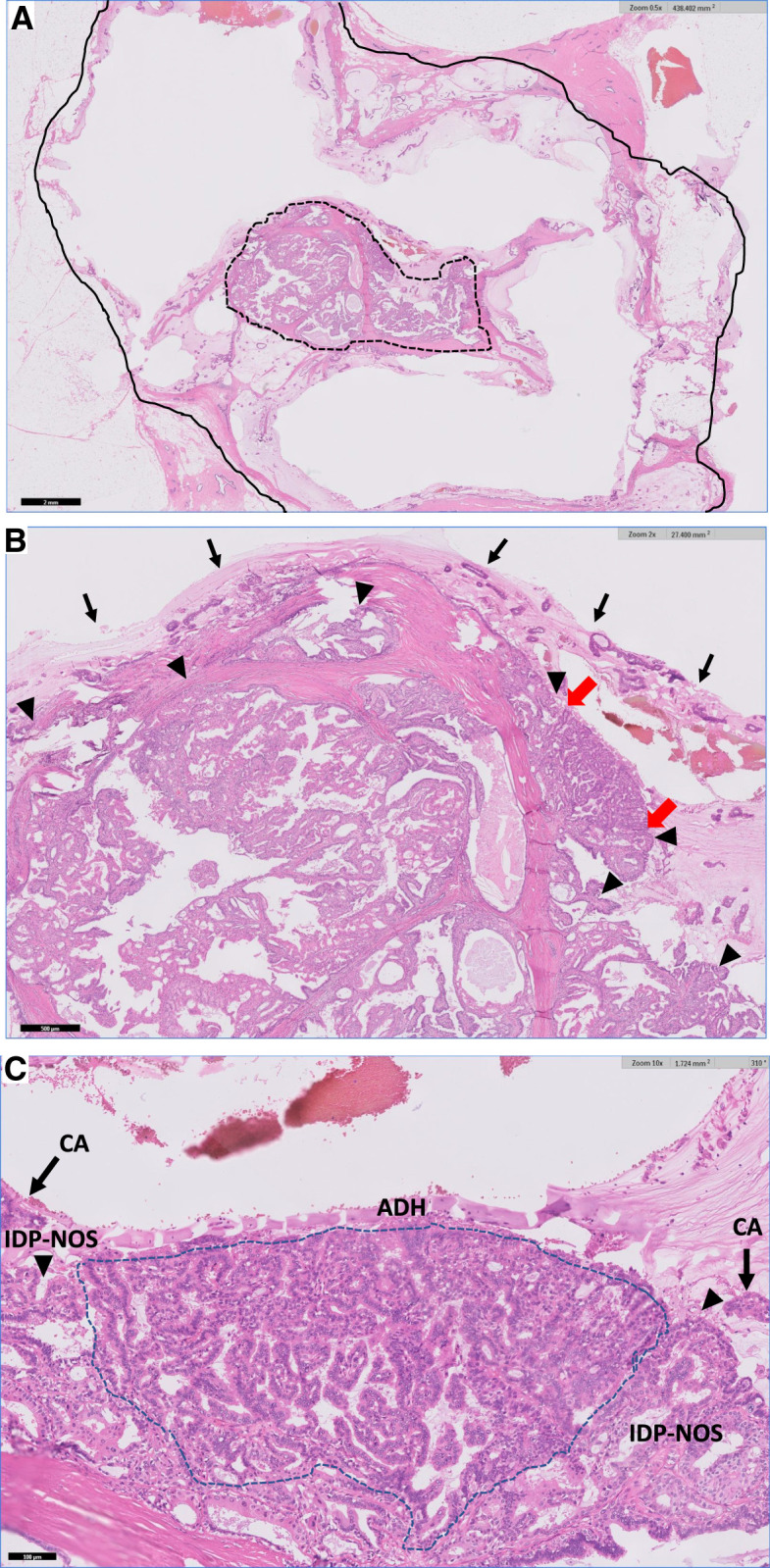
Histology of the resected specimen (hematoxylin and eosin staining). (**A**) Mucinous carcinoma (solid line) has spread to surround the intraductal papilloma with atypical ductal hyperplasia (dashed line). Scale bar = 2 mm. (**B**) Higher-magnification view of a part of 4A. Carcinoma cells (→) are floating in the mucin around the papilloma (▲), which shows ductal hyperplasia (red arrow) Scale bar = 500 μm. (**C**) Higher-magnification view of a part of 4B. A progressive increase in cellular atypia and irregular papillary architecture is observed from the intraductal papilloma (▲) toward carcinoma cells (CA, dashed line). Furthermore, clusters of carcinoma cells are floating within abundant mucin.

**Fig. 5:**
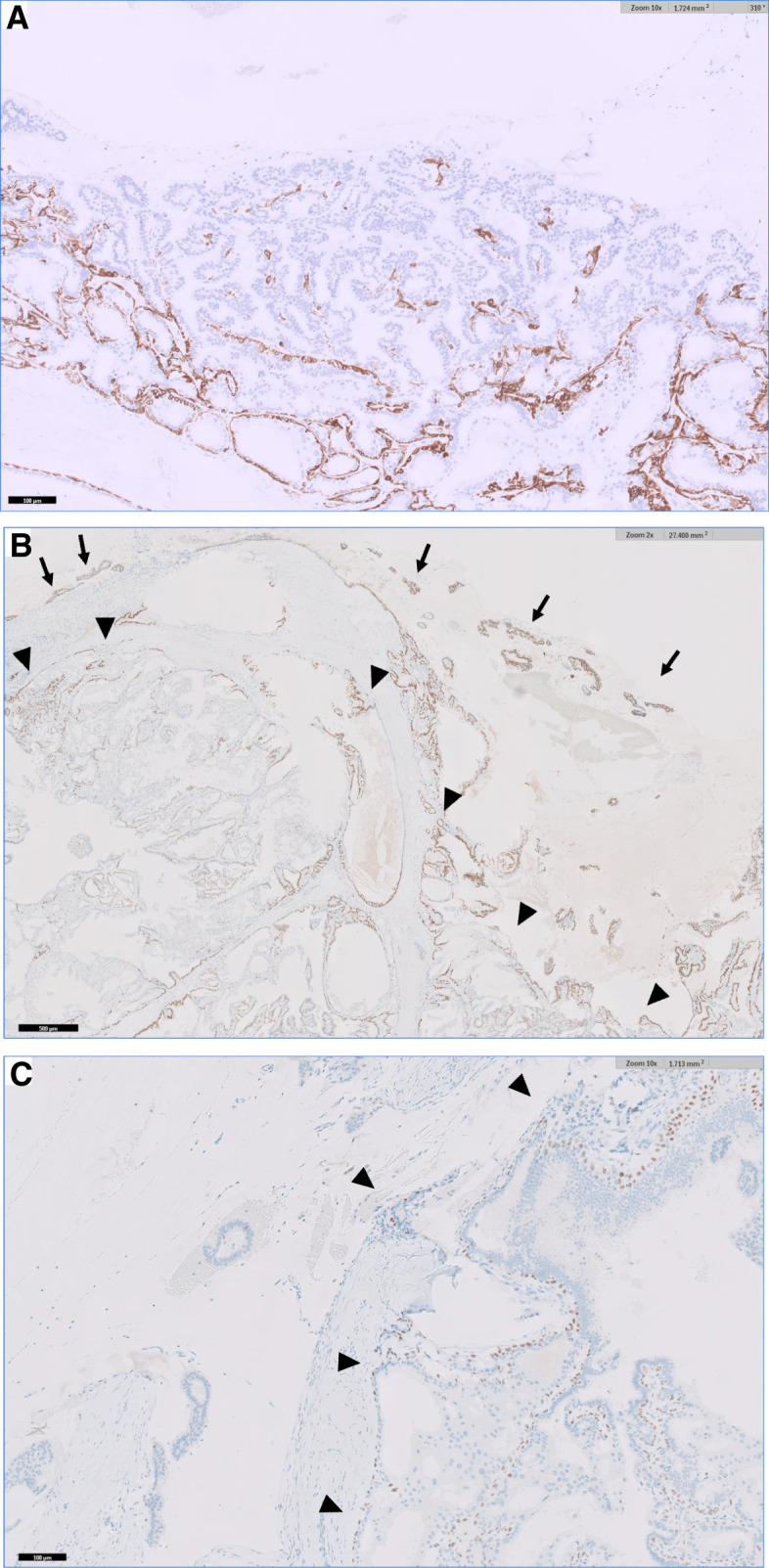
Immunohistochemistry of the tumor lesion. (**A**) The cytoplasm of IDP shows a mosaic-like positive for CK5/6, whereas mucinous carcinoma foci are negative. Scalebar = 2 mm. (**B**) ER is heterogeneously expressed in the papilloma area (▲), but relatively homogeneous in the mucinous carcinoma area (→), shown in the same area as **Fig. 4B**. Scale bar = 500μm. (**C**) IDP (▲) has a continuous myoepithelial lining (p63 positive), whereas myoepithelium is absent in the mucinous carcinoma area. Scale bar = 100 μm.

The patient underwent total mastectomy and sentinel lymph node biopsy for clinical T2N0 left-sided breast cancer. The surgical specimen showed a spread around the papilloma with a distinct transition zone from papilloma to mucinous carcinoma (**Fig. 4A**–**4C**). The specimen exhibited characteristic pathological findings of IDP in the central area, where the epithelium maintained a biphasic appearance, with glandular epithelial and myoepithelial cells displaying papillary proliferation. Clusters of cancer cells floating in mucus lakes surrounded this lesion, exhibiting pathological features consistent with mucinous carcinomas. A gradual increase in cellular atypia, transitioning from benign epithelial cells to carcinoma cells with mucin production, was observed, suggesting a transition zone from IDP to mucinous carcinoma. Cytokeratin 5/6 displayed a mosaic-positive pattern in the papilloma component, indicating a benign pattern, and was negative in the nests of mucinous carcinomas (**Fig. 5A**). ER staining showed 90% positiveity in the mucinous carcinoma component; however, a mosaic pattern of positivity was observed in the papilloma component (**Fig. 5B**). Although p63-positive myoepithelial cells were identified in the papilloma, these were absent in mucinous carcinoma (**Fig. 5C**).

## DISCUSSION

To the best of our knowledge, this is the first report of mucinous carcinoma spreading around a papilloma with a distinct transition zone from the papilloma to the mucinous carcinoma. The coexistence of benign and malignant components with a gradual increase in cellular atypia strongly suggests malignant transformation from IDP to mucinous carcinoma. This case highlights the potential role of IDP as a precursor lesion and underscores the importance of appropriate follow-up even for lesions initially diagnosed as benign.

IDP is a benign intraluminal growth process involving branching fibrovascular cores covered by basal and luminal cells.^4)^ Although generally regarded as nonmalignant, a subset of IDPs may harbor atypical epithelial changes that confer a risk of malignant progression.^7)^ Reported upgrade rates vary, with IDP showing a low but distinct risk, whereas atypical IDP demonstrates a considerably higher potential for malignant transformation.^6)^ Several clinical and radiological features—such as higher BI-RADS category, imaging–pathology discordance, bloody nipple discharge, peripheral location, and lesion size greater than 1 cm—have been proposed as predictors of malignant potential.^7–10)^ When imaging and histological findings are concordant for IDP, the likelihood of upgrade is minimal, with a reported rate of only 1.4%,^11)^ supporting the safety of active surveillance in selected patients.^9–12)^ Various histological transformations have been described in IDP, which may provide morphological clues to its malignant potential. Metaplastic changes, including squamous, apocrine, mucinous, or chondroid differentiation, may occur within IDPs.^5)^ Although direct transition from IDP to mucinous carcinoma has rarely been reported, several observations support the possibility that IDP may serve as a precursor lesion. Solid papillary carcinoma (SPC) has been reported to develop at the site of a prior IDP with ADH showing neuroendocrine differentiation, suggesting a precursor relationship.^13,14)^ Similarly, molecular analyses have revealed that approximately 55% of IDPs share clonal relationships with synchronous DCIS or invasive carcinoma, further supporting their role as precursor lesions.^15)^ Mucinous DCIS is also recognized as a possible precursor of mucinous carcinoma, often showing mucin accumulation within the ducts.^16)^ A previous study identified DCIS in 88 of 130 cases (68%) of mucinous carcinoma, with mucin accumulation in nearly all lesions.^13)^ From a molecular standpoint, recent analyses suggest that a subset of IDPs shares clonal relationships with synchronous DCIS or invasive carcinoma, indicating their potential as true precursor lesions.^15)^ Interestingly, PIK3CA mutations are frequently identified in IDPs but tend to be absent in clonally related cases, implying that distinct molecular pathways may drive benign and malignant evolution.^17)^Another study examining 20 cases of coexisting IDP and DCIS or invasive carcinoma found clonal relationships in 55% (11/20) of cases.^13)^ Taken together, our findings expand the spectrum of IDP-associated malignancies and provide morphological evidence supporting a possible progression pathway toward mucinous carcinoma. In our case, molecular analysis was difficult because only a limited amount of tissue was available; however, if molecular analyses could be performed separately for the areas of IDP-NOS, IDP with ADH, and mucinous carcinoma, it might be possible to demonstrate a sequential progression from IDP-NOS to mucinous carcinoma.

## CONCLUSIONS

We report a case of mucinous carcinoma surrounding an IDP with a distinct transitional zone, strongly suggesting the progression from IDP to mucinous carcinoma. Although IDP is generally regarded as a benign lesion, some cases may harbor malignant potential. Careful follow-up is warranted, and surgical excision should be considered when imaging–pathology discordance is present. These findings highlight the need for risk stratification and for identifying molecular biomarkers to predict which IDPs are likely to undergo malignant transformation.

## DECLARATIONS

### Availability of data and materials

All data from this study are included in this published article.
